# Online Proctoring Put to the Test

**DOI:** 10.1007/978-3-030-65355-2_8

**Published:** 2021-03-20

**Authors:** Colette Cuijpers

**Affiliations:** 1 Tilburg University, Tilburg, The Netherlands; 2 Tilburg University, Tilburg, The Netherlands; 3 Tilburg University, Tilburg, The Netherlands; 4 Tilburg University, Tilburg, The Netherlands; Department of Law, Technology, Markets, and Society (LTMS), Tilburg Law School, Tilburg, The Netherlands

## Abstract

Traditionally, universities are places where students and professors physically interact. Knowledge and skills of students are generally tested in oral and written forms, on campus. In times of COVID-19, however, a new common in education is called for as students are unable to be physically present on campus. Both education and assessments need to take place at a distance. This development creates opportunities. However, education at a distance also bears risks, such as the risk of fraud. Universities are responsible for safeguarding the quality of education, assessments, and diplomas. Only students who actually master the learning objectives should receive a diploma. Online proctoring systems can help to achieve this. A challenging issue is that some students oppose these systems from the perspective of privacy and data protection. In view of the key focus of this book, moving towards a new common while preserving the best of the old common, it is thus important to put online proctoring systems to the test and consider their impact on human rights.

Traditionally, universities are places where students and professors physically interact. Knowledge and skills of students are generally tested in oral and written forms, on campus. In times of COVID-19, however, a new common in education is called for as students are unable to be physically present on campus. Both education and assessments need to take place at a distance. This development creates opportunities. It becomes easier and cheaper for students to participate in Bachelor’s and Master’s programs across the globe. Moreover, the flexibility of online education also offers opportunities for students who want to combine their studies with a job.

However, education at a distance also bears risks, such as the risk of fraud. Universities are responsible for safeguarding the quality of education, assessments, and diplomas. Only students who actually master the learning objectives should receive a diploma. Online proctoring systems can help to achieve this. Online proctoring is surveillance at a distance, for which many different systems are available. Most of these systems can be characterized by several common functionalities: detecting and disabling computer functionalities such as copy-paste and downloading, taking images off and recording both screen and student, and analyzing the gathered data to signal irregularities that may indicate fraud. A challenging issue is that some students oppose these systems from the perspective of privacy and data protection. In view of the key focus of this book, moving towards a new common while preserving the best of the old common, it is thus important to put online proctoring systems to the test and consider their impact on human rights.

## Privacy: The Need for a Fair Balance

The right to private life is vested in Article 8 of the European Convention on Human Rights (ECHR): “Everyone has the right to respect for his private and family life, his home and his correspondence.”^1^ The second paragraph of Article 8 makes it clear that infringements of privacy can be justified if certain criteria are met. These criteria are “in accordance with the law” and “in the interests of national security, public safety or the economic well-being of the country, for the prevention of disorder or crime, for the protection of health or morals, or for the protection of the rights and freedoms of others.” Moreover, the interference must be “necessary in a democratic society.”

Universities have a legal duty to provide education, assessments, and diplomas. This is important for the economic well-being of a country and in the interest of the right to education (Article 2 of Protocol No. 1 ECHR). The crux of the privacy test is thus whether online proctoring is necessary for a democratic society. From case law, it becomes apparent that this test concerns the reasonableness of the infringement in relation to the social interest it serves (Council of Europe 2019). This is assessed on the basis of the principles of proportionality and subsidiarity. Proportionality requires that the measure—online proctoring—can achieve the purposes of identification and fraud prevention. This seems to be the case in view of the functionalities of online proctoring systems. The principle of subsidiarity requires an assessment whether these aims can be achieved in a less privacy-invasive manner. This means careful consideration must be given to the types of assessment that justify online proctoring, as well as the functionalities of such a system. For many exams, alternative options are available, such as papers, oral exams, and take-home exams. Online proctoring only seems necessary for large-scale exams of great importance, consisting of multiple-choice questions and closed questions at the level of remembering and understanding (Surf 2020).

Monitoring the screen, disabling certain functionalities, or detecting second screens do impact a student’s private sphere. However, these measures seem necessary and justified as during an exam these functionalities are not allowed either. If restricted for the duration of the exam, there is no unjust breach of privacy. For recordings and images, I am not entirely convinced. Scanning the entire room seems unnecessary, only showing the workspace is sufficient.^2^ But what about continuous monitoring: is this actually required? Are several snapshots sufficient to verify identity and detect fraud, or still images combined with a blurred video stream? To satisfy the principle of subsidiarity, and thus to create a fair balance between the need to detect fraud and the right to privacy, the least invasive settings of the system should be applied.

For fraud detection, several alternatives are available to online proctoring such as plagiarism checks and randomization of exam questions. Alternatives for verifying the identity of a student at a distance are less obvious. The only way to really check the identity of a student at a distance is visibility via a webcam, as login credentials can easily be shared. There certainly is a valid reason to check the student’s identity during exams. The question that arises is whether the identity of students is sufficiently safeguarded with alternative assessment methods, such as take-home exams and papers. If this is not the case, what does this mean for the argument that it is necessary for online-proctored exams? In my opinion, this is a problem that needs to be further investigated, not only from the perspective of privacy but also from the perspective of safeguarding the quality of education.

## Lawful Processing of Personal Data

In the EU, the General Data Protection Regulation (GDPR) contains the rules on how to process personal data. Noncompliance can lead to high fines. The main principles for processing of personal data—any information relating to an identified or identifiable natural person (Article 4 (1) GDPR)—can be found in Articles 5 and 6 of the GDPR.^3^ Article 5 states that personal data can only be processed if several principles are taken into account: the processing is based on a specified purpose, no more data may be processed than necessary to achieve that purpose, data must be of good quality, it must be transparent why and how personal data are being processed, data must be properly secured, data subjects must be granted certain rights—such as access, rectification, and erasure—and the data controller must demonstrate that he/she adheres to these principles, also known as the principle of accountability. Online proctoring is only possible if these principles are taken into account. Moreover, Article 6 of the GDPR requires a legitimate ground for the processing of personal data. Universities can base online proctoring on the ground “necessary to perform a public task.” A Dutch court recently ruled this ground applicable as, by law, universities are required to guarantee the quality of education, assessments, and diplomas.^4^ What could be relevant in relation to online proctoring is that the GDPR only allows the processing of special categories of data on the basis of consent.^5^ It might very well be that such data are being processed with online proctoring. Even though the Dutch court concluded this not to be the case, the reasoning of the court is very limited and inconclusive. The court merely states no biometric data are processed in online proctoring. However, the GDPR definition of biometric data explicitly refers to facial images. These are being processed by online proctoring systems.

If this leads to the conclusion that online proctoring is only allowed on the basis of consent, a problem arises. According to the GDPR, consent needs to be freely given (Article 7 GDPR). In a hierarchical relationship—such as between university and student—consent is not freely given if no real opt-out possibility exists, meaning that the student can decline taking part in the online proctoring exam. This requires the university to offer students an assessment alternative to online proctoring—e.g., an oral exam—without there being any negative consequences for the student, such as study delay.

## Conclusion

When exams on university premises are impossible, online proctoring can be a valid alternative if compliant with the rights to privacy and data protection. From a privacy perspective, the key question concerns subsidiarity: are there other less invasive alternatives available for a specific assessment? To answer this question, both the type of assessment and the functionalities of the proctoring system are relevant to consider. From the perspective of data protection, the main question seems to be the need for consent when processing facial images for identification purposes and the problem of freely given consent in hierarchical relationships. For the time being, the safest approach is online proctoring as an option. Offering an equal alternative to online proctoring—without negative implications for students who want to opt-out—meets the “freely given” requirement of consent and the principle of subsidiarity.

## Footnotes

1 European Convention on Human Rights (ECHR) (2020).

2 This was also concluded in a Dutch court case regarding online proctoring. Court Amsterdam, 11-06-2020. C/13/684665/KG ZA 20-481, ECLI: NL: RBAMS: 2020: 2917.

3 General Data Protection Regulation (GDPR) (2020).

4 Court Amsterdam (2020)

5 Article 9 GDPR states “Processing of personal data revealing racial or ethnic origin, political opinions, religious or philosophical beliefs, or trade union membership, and the processing of genetic data, biometric data for the purpose of uniquely identifying a natural person, data concerning health or data concerning a natural person’s sex life or sexual orientation shall be prohibited.”
